# Doctor’s Preference in Providing Medical Service for Patients in the Medical Alliance: A Pilot Discrete Choice Experiment

**DOI:** 10.3390/ijerph17072215

**Published:** 2020-03-26

**Authors:** Richard Huan Xu, Lingming Zhou, Yong Li, Dong Wang

**Affiliations:** 1 Jockey Club School of public health and primary care, the Chinese University of Hong Kong, Hong Kong, China; richardhxu@cuhk.edu.hk; 2 School of Health Management, Southern Medical University, Guangzhou 510515, China; peterlm_87@hotmail.com (L.Z.); yongli1995@126.com (Y.L.)

**Keywords:** medical alliance, healthcare, doctor preference

## Abstract

This cross-sectional survey study explored whether doctors in Guangdong, China preferred to provide extra healthcare services within the context of their medical alliances (MAs). Specifically, a discrete choice experiment (DCE) was conducted to investigate whether doctors preferred to provide extra services at low-tier hospitals within their MAs. A literature review, focus group interview, and expert group discussion resulted in three main attributes (i.e., working time, income, and hospital location) and corresponding levels, which were combined to create 24 profiles that were randomly presented to participants. A conditional logit model was then employed to calculate utility scores for all profiles. A total of 311 doctors completed the DCE questionnaire. The coefficients for each level within the three attributes were ordered and found to be statistically significant. Working time had the greatest influence on utility scores, increasing by one hour per week (beta = 1.4, odds ratio (OR) = 4.07, *p* < 0.001), followed by income, which increased by 30% per month (beta = 1.19, OR = 3.3, *p* < 0.001). The utility scores for all profiles ranged between −0.27 and 3.07. Findings indicated that participants made trade-offs with respect to providing extra services within their MAs. Furthermore, utility varied between different subpopulations.

## 1. Introduction

*Kanbing nan* and *kanbing gui* (that is, too inaccessible and too expensive to visit the doctor) are the sequelae of China’s painful sociopolitical transition. Even after years of making efforts to improve conditions, the nation’s fragmented healthcare system is still negatively affected by the lack of efficient service deliveries [1]. In 2015, the State Council of China issued new guidelines for building an innovative tiered health-care delivery system to replace the existing hospital-centric pattern [2]. This new system entailed that the roles of medical institutions (especially public hospitals) should be clarified based on their designated functions at different levels. Further, new rules mandated that resources and diagnosis results would be shared while ensuring that integrated care was available across medical service levels [3].

The central Chinese government has since tiered the delivery of healthcare services through a new form of organization called the *Yiliao lianheti* (medical alliance; MA) [2,4]. MAs are not a mainstream part of the western health care industry. They take many forms, but mainly operate within the private healthcare system or at the community-level of public healthcare, and primarily function to supplement healthcare services [5,6]. In China, MAs usually consist of three tiers. The top-tier is the leading hospital, which is typically a national or provincial level institution that functions as a regional healthcare center. The second tier is the city or district level hospital, while the bottom-tier is the county level healthcare service provider (i.e., county or town level clinic/hospital). The second and bottom tier members are conceptually combined to form a lower-tier (primary-level) hospital for the purpose of daily operations. The leading hospital typically manages the MA while providing guidance and support to the lower-tier hospitals and offering an accessible way to make referrals [7]. One of the pillars supporting MA operation is the ability to send medical professionals (especially senior specialists) from leading hospitals to work with lower-tier members within the MA. In this capacity, they periodically provide training, guidance, and direct medical care services [8]. Although the political design of this system seems to provide important support for improving service quality at relatively low costs, it ignores the reality, in which highly burdensome workloads are presented to doctors at the tertiary-level hospitals. Further, incomes are closely related to these workloads, there is limited motivation and encouragement for doctors, and specialists must provide extra services while visiting lower-tier MA members [9]. A recent policy report by the State Council identified that doctors’ attitudes toward MA-related work were essential for service quality and patient satisfaction [10]. However, we know of no previous empirical studies on this important topic. In 2018, the State Council of China announced that it would establish an MA system throughout the country over a period of a few years. It is therefore crucial to understand and include doctors’ preferences in the political framework design, thus ensuring sustainable MA development. As such, this study conducted a preliminary exploration of doctors’ preferences for providing medical services within the MA through a discrete choice experiment (DCE).

## 2. Materials and Methods

### 2.1. Discrete Choice Experiment

The DCE technique is commonly used in the health economics field [11]. It is rooted in Lancaster’s theory of consumer choice and random utility theory [12]. A DCE questionnaire entails that two or more alternatives are shown to the respondent, who is then asked to choose the one they prefer for maximizing their own utility. The alternatives are usually combinations of the levels and attributes; these attributes represent the essential characteristics of the targeted good or service. By presenting respondents with multiple scenarios, DCEs can successfully be used to determine which attributes drive their preferences and reveal any trade-offs respondents are willing to accept between attributes [13]. There are several advantages to the DCE. For example, it presents an affordable and quick way of investigating the relevance of potential policy and/or service options [14]. On the other hand, the methodology is relatively complicated, meaning that researchers require advanced knowledge of the experimental design and must be able to use statistical software [15]. Considering that a range of factors may affect a given health professional’s motivations to provide medical services within the MA [12,16,17], it is possible to investigate the influences of different factor combinations on their choices. As such, this study adopted the DCE to explore doctors’ preferences for different service profiles within the MA.

### 2.2. Setting

This study was conducted in Guangdong (GD) province, which is located in southern China and contains a population of more than 110 million. GD is also the largest economy within China. In 2017, gross health expenditures were 461.9 billion RMB (~67 billion US dollars), accounting for 5.2% of the GDP. GD has 734 public and 818 private hospitals, with 0.28 and 0.34 million registered doctors and nurses, respectively, in 2018. These professionals provided nearly 840 million healthcare services per person in 2018 (www.gdhealth.net.cn). Further, nine public hospitals in GD were included in the Top-100 China hospital in 2018. Moreover, the Luohu model (a representative MA model that is popular in urban areas) was initiated and developed in GD [8].

### 2.3. Attributes and Levels

The first step of the DCE process is to identify and develop the attributes and corresponding levels. Based on reviews of the literature and government reports on the barriers and facilitators to motivating doctors who work in primary healthcare settings [2,4,9,10,18,19,20,21,22,23,24,25,26,27,28], this study identified the nine following attributes that may influence doctors’ working preferences within the MA: a) income, b) hospital location, c) working time, d) type of contract, e) training and career development, f) working environment, g) policy, h) benefits package, and i) workload. This list was then submitted to an expert panel comprised of doctors, researchers, and administrators for discussion. As such, the “type of contract” and “training and career development” attributes were removed, while the “hospital location and working environment” and “income and benefits package” attributes were combined to create the two new attributes of “location of working hospital” and “increment in income per month,” respectively. Further, the “working time” attribute was revised to “increment in working time per month.” Then, a total of seven focus group interviews (including 22 doctors and 17 nurses) were conducted in five cities in GD (Guangzhou, Shenzhen, Zhanjiang, Meizhou, and Shaoguan). These were designed to discuss the remaining attributes and levels until a consensus was reached. The “policy” attribute was ultimately removed based on these interviews. However, all participants agreed that “income,” “working time,” and “hospital location” were three key concepts that influenced medical professionals when deciding whether to accept or reject the service plans provided by their hospitals when asking them to work with low-tier members within the MA. Because no previous DCE studies had focused on this topic in China, we welcomed suggestions to add any new attributes to the list for open discussion during the group interviews. However, none were added.

After concluding the focus group interviews, the three attributes and three accompanying levels for each were returned to the expert panel for a final review. A subsequent discussion and review of national and regional documents, briefings, and reports resulted in a revision in which the “increment in working time per month” attribute was changed to “increment in working time per week,” which was believed to more accurately reflect Chinese policy. Then, a draft of the three attributes (including two with five levels and one with three levels) was confirmed. In order to evaluate attribute validity, this draft was provided to five groups of patients who gave their opinions about the potential factors associated with doctors’ working preferences from the customer’s perspective. No further revisions were needed based on this process. Finally, the research team reviewed all information and evidence from each of the previous steps. They then created a study questionnaire containing the three following attributes: “increment in working time per week” (TI), “increment in income per month” (IN), and “location of working hospital” (LO), each with corresponding levels (Table 1).

### 2.4. Experiment Design

A full factorial design using the abovementioned attributes and levels resulted in 5^2^ * 3 = 75 possible profiles. This means that there were 2775 possible pairwise choice sets that could be selected. Using the R software, 24 pairwise choice sets were thus constructed from the 75 profiles using a D-optimality algorithm [6]. The mechanism of the D-optimality criterion is designed to minimize the joint confidence sphere around the estimated model parameters based on a maximization of the determinant of the inverse of the variance–covariance matrix in the maximum-likelihood estimation [29]. In this study, the D-efficiency value was 93.87, which suggests an acceptable design. The design only allowed for checking main effects. All of the choice sets were checked for plausibility, and no unreasonable choices sets were included. There is little guidance on the optimal number of DCE tasks for each respondent to complete in a single survey. To reduce the cognitive burdens presented to respondents, based on the suggestions by previous systematic review [30], we opted to organize the 24 choice sets into two blocks of 12 choices. Participants were randomized so that each received one of the two DCE questionnaire versions. An example of the DCE choice set is given in Table 2. Each choice set was composed of two alternatives (profiles) (i.e., Plan A and Plan B). These profiles offered plans containing the three attributes, but given at certain levels.

### 2.5. Survey and Data Collection

Data were collected from the first GD plenary conference held for MAs in December 2019. All participants were doctors or managers who belonged to different MAs operating in GD. The conference aimed 1) to develop the GD association of MAs, 2) vote for the first central committee of the GD association of MAs, and 3) hold the first academic symposium about the development and the sustainability of MAs in GD. A team of well-trained investigators who had substantial experience with the face-to-face survey method was invited to conduct the survey. All symposium attendees were invited to participate. Here, participants were required to complete a two-page questionnaire which contained two parts. The first collected participant demographic characteristics, including working status and other socioeconomic information, while the second contained the DCE questionnaire. Participants were first required to complete a DCE task with a dominant alternative to evaluate the validity of the study. All participants were invited to complete their questionnaires prior to the start of the symposium or negotiated another time to do so after the symposium. All survey purposes and instructions related to the DCE were given by the investigators before the start of the survey. The survey took approximately 10–15 min to complete for each participant. All participants completed consent to participate forms and all responses were anonymous. This study was approved by the institutional review board of the third affiliated hospital of Southern Medical University.

### 2.6. Statistical Analysis

Descriptive statics were reported for respondent demographics, socioeconomic status, and working status. Choice data were modeled using a random utility maximization framework. The outcome variable was binary choice data, and attributes were dummy coded. A conditional logit model (CLM) was adopted to conduct the statistical analysis. The specific CLM was introduced by McFadden [31] and was based on the random utility theory. In this study, choice data from a two-alternative DCE were analyzed using a limited depend-variable model. Here, 1 represented the chosen alternative, while 0 represented the alternative that was not chosen. The CLM provides a basic method for analyzing data generated by the DCE. According to random utility theory, the utility (U) associated with a particular chosen plan consisted of two components, including an observable component V and unobservable error (ε). The theory assumes that a person who is presented with a choice between two alternatives will choose the option that maximizes their utility [32].

The basic form of the model estimation was as follows

U_n_ = V_n_ + ε_n_

In this study, the component of Vn can further explained, as follows

V_n_ = ß_0_ + ß_1_* TI _4h_ + ß_2_ * TI _3h_ + ß_3_* TI _2h_ +ß_4_* TI _1h_ + ß_5_* IN _15%_ +ß_6_* IN _20%_ + ß_7_ * IN _25%_ + ß_8_* IN _30%_ + ß_9_* LO _Suburb_ + ß_10_ * LO _Downtown_

## 3. Results

A convenient sample of 311 participants was utilized. All participants completed the questionnaire, with 300 (96.4%) indicating that it was easy to understand. Based on the demographic section, 61.09% of respondents were male, while nearly 37% were senior doctors. Further, average age and working years were 38.86 and 11.91, respectively. After randomization, 143 participants completed the first version of questionnaire, while 168 completed the second version. There were no statistically significant differences in demographic characteristics between respondents who completed the different versions (Table 3).

Table 4 presents the main effects of the CLM. As shown, the coefficients of each level were ordered as expected and found to be statistically significant. TI increased by one hour per week (beta = 1.4, odds ratio [OR] = 4.07, *p* < 0.001), leading to the maximum incentive for the utility score. Next, IN increased by 30% per month (beta = 1.19, OR = 3.3, *p* < 0.001). As opposed to working in county locations, participants preferred to work in hospitals located in downtown (beta = 0.75, OR = 2.11, *p* < 0.001) and suburban (beta = 0.31, OR = 1.36, *p* < 0.001) areas.

Table 5 shows the utility scores for all attribute/level combinations based on the main effect model for the selection of the top and bottom 10 profiles, respectively. As shown, utility scores ranged from −0.27 to 3.07. Variations in the utility scores between profiles indicated the impacts of the changes on the relative values for different profiles, thus showing participant selections for the most and least desirable combinations of attributes/levels. Table 5 also shows the estimated probability of choosing one profile over the others. The top-ranked profile had a 5.37% probability of being chosen, while the bottom-ranked profile had a 0.19% probability of being chosen. 

Table 6 and Table 7 show the results of selective subgroup analyses conducted using the CLM. As seen, profiles with few extra working hours or requiring participants to work in downtown areas contributed to a higher utility score for female and senior doctors than that derived for male and junior doctors.

## 4. Discussion

This study was the first to implement a DCE designed to investigate doctor preferences for providing medical services within MAs in China. We found that all three presented attributes (i.e., the monetary (IN) and non-monetary (TI and LO) factors) significantly influenced which service plans doctors preferred. Further, possible heterogeneity was found in regard to sex and professional rank.

We specifically found that the influence of location was important for the utility of doctors. Reviewing the utility scores, four of the five highest ranked profiles all involved working locations in downtown areas. In China, there are substantial gaps in economic development between urban and non-urban areas. As such, there are also pronounced differences in working conditions, medical infrastructures, and patient literacy levels, all of which negatively influence doctors’ preferences for working in those areas [9]. Indeed, it is difficult to encourage medical professionals to work in non-urban areas throughout the world. For example, McGill et al. found a significantly low likelihood that doctors would establish careers in rural areas in Australia [26]. Further, Rechel et al. pointed out that it was highly challenging to attract highly skilled doctors to work in remote locations in eight high-income countries [28]. Moreover, while profiles that included work in urban areas contributed to higher utility scores for male doctors, we found that the coefficient for males was smaller than that for females in the main effect model. This is congruent with previous studies finding that fewer female doctors worked in remote areas [21,25]. When compared to senior doctors, junior doctors also seemed to be less concerned that their utility was affected based on hospital location. These findings suggest that policy strategies and public incentives should reflect doctors’ preferences for working location while considering both gender and professional rank.

As expected, participants preferred to spend fewer hours working at low-tier hospitals. In China, most tertiary-level hospitals are understaffed and overwhelmed, with 91.8% of doctors being required to work overtime [33]. These doctors thus experience considerable work-related tiredness, fatigue, and cognitive impairment due to longer working hours [34]. In the UK, working time regulations have significantly reduced these burdens [20]. However, a lack of empirical evidence on the relationships between working time and negative experiences in the medical profession impede such legislation in China. Outside of using objective indicators to calculate working hours, our findings suggest that subjective utility may play an equally or more important role in how doctors make their working decisions. For example, we found that a small discrepancy in disutility was generated between 2 h and 3 h; this may be offset if other incentives are applied. We also made a notable finding when assessing the profiles for the top 10 highest utility scores. That is, when decision makers were provided with a 30% increment in income and ensured that their working hospital would be located in a downtown area, then the utility generated for doctors through the 3 h plan was not any lower than that generated by some other 1 or 2 h plans. Nonetheless, given the well-documented detrimental effects of long working hours on health in the medical profession [34], utility calculations should also consider other health-related attributes.

We also found that profiles with income increments of 30% and 25% were more likely to be chosen than otherwise identical profiles with lower income increments. This indicates that financial incentives are still some of the most important factors for increasing doctors’ working utility within their MA. From 2008 to 2012, nearly 80% of doctors working in China resigned from county-level hospitals and moved to city-level hospitals, with income discrepancies between urban and rural areas being the main reason [35]. The impact of income on medical talent shortages in the primary care setting is also a problem in Western countries [17,36]. This study’s quantification of financial incentives revealed only small differences in utility between 20% and 25% income incentives. However, the difference between 20% and 15% was very substantial, thus indicating that financial incentives have varied and non-linear impacts on utility. As such, working efficiency may be improved through marginal increments in subjective utility rather than continual increases in absolute income amounts. Additionally, while we found that income increments contributed to higher utility scores for senior doctors than junior doctors, this statement should be taken with caution considering that few senior doctors participated in the survey.

MAs provide cost-effective opportunities to maintain public health during new challenges presented by aging populations and the increasing prevalence of chronic disease [37]. Further, it is difficult to encourage medical professionals to work in primary care throughout the world. Here, MAs may provide valuable alternatives, especially in developing countries that need to review and rebuild their healthcare systems. This study adopted the DCE method to elicit doctors’ preferences in this regard. While DCEs are increasingly used in the health care and economics fields [38], they are insufficiently used in the context of healthcare in China. Due to the exploratory nature of this study, we intended to maintain a basic experimental design. As such, we only included attributes that were most directly known to significantly affect doctors’ preferences. The findings presented here are based on 75 profiles designed to compare the effects of trade-offs doctors make between different attributes in regard to their utility scores. These results can help inform decision makers about attribute/level combinations that may be more or less preferred at the societal level. Further, MA leaders can leverage the utility scores created through this analysis to adjust service plans and maximize efficiency. Taken as a whole, this study’s findings suggest that doctors give higher priority to certain combinations of working time, income, and working location. The utility score may provide another person-centered perspective for evaluating the effectiveness of services provided by MAs.

There are also several limitations that should be addressed. First, our experimental design was not complex and the results presented were produced through a simple analytical technique. Second, respondents were recruited at a professional conference, which creates some concern about our findings. That is, although they were from different regions of GD and had different specialties, we assumed that all respondents would hold positive attitudes toward the MA concept. As such, the preferences of doctors with non-positive attitudes may be underestimated in this regard. A substantial degree of selection bias may also have been caused by these limitations. Third, the sample size may have been insufficient for detecting preference heterogeneities through the DCE method. Results may therefore be of limited generalizability. A larger sample is thus needed to verify our findings. Fourth, our intention to keep the DCE at a basic level meant that other attributes were not included. These should be explored in future experiments. Fifth, the cross-sectional nature of the survey design prevented the establishment of causal relationships. Lastly, given the limited sample size, the effects of interaction were not considered in the analysis and should thus be included in future studies.

## 5. Conclusions

This study’s findings provide empirical evidence that doctors make trade-offs between income, working time, and working location with respect to providing extra services within their MAs. Working plans should thus be provided based on the preferences of medical professionals and designed to maximize their utility. Further, a potential heterogeneity of preferences was found among participants in terms of gender and professional rank. These factors should also be considered when developing political frameworks. Future studies should refine the attributes/levels implemented in this study, while new surveys should be conducted among larger and more representative samples to further explore marginal changes that may thus be revealed in the preferences of doctors and other medical professionals. Finally, researchers should attempt to determine how these preferences interact.

## Figures and Tables

**Table 1:** The attribute and levels of the discrete choice experiment (DCE).

Attribute	Level
Increment in working time per week	1 h more 2 h more 3 h more 4 h more 5 h more
Increment in the income per month	10% more 15% more 20% more 25% more 30% more
Location of working hospital	County Suburb Downtown

**Table 2:** The sample of choice set.

	Plan A*	Plan B
Increment in working time per week	5 h more	2 h more
Increment in the income per month	30% more	15% more
The location of working hospital	suburb	downtown
WHICH PLAN YOU PREFER	□	□

* The questions in the survey were presented in Chinese.

**Table 3:** The background information of respondents.

	n = 311	% = 100	Questionnaire			
			Ver1	Ver2	χ^2^/F-Value	*p*-Value
Sex						
Male	190	61.09	83	107	1.04	0.309
Female	121	38.91	60	61		
Educational level						
Bachelor	149	48.38	71	78	5.98	0.051
Master	105	34.09	54	51		
Ph.D	54	17.53	17	37		
Medical career grades						
Resident	90	28.94	48	42	6.26	0.1
Resident specialist	105	33.76	52	53		
Associate consultant	80	25.72	29	51		
Consultant	36	11.58	14	22		
Ward’s leader						
Yes	132	43.28	53	79	3.09	0.08
No	173	56.72	87	86		
Income						
Lower than average	148	47.59	72	76	0.85	0.65
Equal to average	139	44.69	61	78		
Higher than average	24	7.72	10	14		
						
Age (mean, sd)	38.86	8.83	36.03	37.57	2.35	0.126
Working years (mean, sd)	11.91	8.82	11.34	12.37	0.96	0.328

**Table 4:** Main effects model results.

Attribute Levels	Beta	Std. Err.	OR	95% C.I.	*p*-Value
Constant (ß0)	−0.27	0.04			<0.001
Working time					
4 h (ß_1_)	0.46	0.09	1.58	1.32, 1.88	<0.001
3 h (ß_2_)	0.70	0.08	2.02	1.72, 2.39	<0.001
2 h (ß_3_)	1.01	0.09	2.75	2.23, 3.32	<0.001
1 h (ß_4_)	1.40	0.09	4.07	3.42, 4.85	<0.001
Monthly income increment					
15% (ß_5_)	0.29	0.07	1.33	1.16, 1.54	<0.001
20% (ß_6_)	0.80	0.08	2.29	1.9, 2.64	<0.001
25% (ß_7_)	0.89	0.07	2.44	2.1, 2.83	<0.001
30% (ß_8_)	1.19	0.11	3.30	2.64, 4.14	<0.001
Working place					
Suburb (ß_9_)	0.31	0.08	1.36	1.16, 1.58	<0.001
Downtown (ß_10_)	0.75	0.09	2.11	1.73, 2.56	<0.001

Std. Err.: Standard error of coefficient; OR: Odds ratio; 95% C.I.: 95% Confidence interval of OR

**Table 5:** DCE model: estimated utility score by selected scenario based on main effect model.

	Scenario Description (Attributes/Levels)					
Ranked by Probability	Time Increment	Income Increment	Working Place	Utility	Probability (%)	Cumulative Probability (%)
1	1 h	30%	Downtown	3.07	5.37	5.37
2	1 h	25%	Downtown	2.77	3.98	9.35
3	1 h	20%	Downtown	2.68	3.64	12.99
4	2 h	30%	Downtown	2.68	3.64	16.63
5	1 h	30%	Suburb	2.63	3.46	20.09
6	2 h	25%	Downtown	2.38	2.69	22.78
7	3 h	30%	Downtown	2.37	2.67	25.45
8	1 h	25%	Suburb	2.33	2.56	28.02
9	1 h	30%	County	2.32	2.54	30.55
10	2 h	20%	Downtown	2.29	2.46	33.02
						
66	5 h	20%	County	0.53	0.42	97.05
67	4 h	10%	Suburb	0.5	0.41	97.46
68	4 h	15%	County	0.48	0.4	97.86
69	5 h	10%	Downtown	0.48	0.4	98.26
70	3 h	10%	County	0.43	0.38	98.65
71	5 h	15%	Suburb	0.33	0.35	98.99
72	4 h	10%	County	0.19	0.3	99.30
73	5 h	10%	Suburb	0.04	0.26	99.56
74	5 h	15%	County	0.02	0.25	99.81
75	5 h	10%	County	-0.27	0.19	100.00

**Table 6:** Sub-group effect model stratified by sex.

Attribute Levels	Beta	Std. Err.	OR	95%C.I.	*p*-Value
**Male**					
Constant (ß_0_)	−0.28	0.06			<0.001
Working time					
4 h (ß_1_)	0.27	0.11	1.31	1.05, 1.65	0.017
3 h (ß_2_)	0.46	0.11	1.59	1.3, 1.95	<0.001
2 h (ß_3_)	0.74	0.12	2.11	1.67, 2.66	<0.001
1 h (ß_4_)	1.13	0.11	3.10	2.48, 3.86	<0.001
Monthly income increment					
15% (ß_5_)	0.29	0.09	1.28	1.07, 1.54	<0.001
20% (ß_6_)	0.77	0.11	2.15	1.75, 2.66	<0.001
25% (ß_7_)	0.91	0.09	2.49	2.05, 3.03	<0.001
30% (ß_8_)	1.16	0.15	3.19	2.41, 4.26	<0.001
Working place					
Suburb (ß_9_)	0.15	0.09	1.16	0.96, 1.42	0.124
Downtown (ß_10_)	0.43	0.12	1.53	1.21, 1.95	<0.001
**Female**					
Constant (ß_0_)	−0.27	0.07			<0.001
Working time					
4 h (ß_1_)	0.76	0.15	2.13	1.6, 2.83	<0.001
3 h (ß_2_)	1.10	0.16	3.02	2.32, 3.94	<0.001
2 h (ß_3_)	1.48	0.14	4.37	3.22, 5.93	<0.001
1 h (ß_4_)	1.89	0.15	6.63	4.9, 8.94	<0.001
Monthly income increment					
15% (ß_5_)	0.35	0.12	1.42	1.13, 1.79	0.003
20% (ß_6_)	0.89	0.14	2.43	1.84, 3.22	<0.001
25% (ß_7_)	0.90	0.13	2.47	1.93, 3.16	<0.001
30% (ß_8_)	1.30	0.19	3.67	2.53, 5.31	<0.001
Working place					
Suburb (ß_9_)	0.61	0.14	1.84	1.4, 2.41	<0.001
Downtown (ß_10_)	1.33	0.17	3.78	2.69, 5.26	<0.001

Std. Err.: Standard error of coefficient; OR: Odds ratio; 95% C.I.: 95% Confidence interval of OR.

**Table 7:** Sub-group effect model stratified by professional rank.

Attribute Levels	Beta	Std. Err.	OR	95% C.I.	*p*-Value
**Junior staff**					
Constant (ß_0_)	−0.24	0.05			<0.001
Working time					
4 h (ß_1_)	0.54	0.11	1.72	1.38, 2.16	<0.001
3 h (ß_2_)	0.86	0.10	2.21	1.8, 2.69	<0.001
2 h (ß_3_)	1.02	0.12	2.76	2.2, 3.49	<0.001
1 h (ß_4_)	1.35	0.11	3.87	3.1, 4.81	<0.001
Monthly income increment					
15% (ß_5_)	0.25	0.09	1.28	1.08, 1.52	0.005
20% (ß_6_)	0.73	0.11	2.08	1.68, 2.59	<0.001
25% (ß_7_)	0.86	0.09	2.35	1.95, 2.83	<0.001
30% (ß_8_)	1.09	0.14	2.98	2.25, 3.97	<0.001
Working place					
Suburb (ß_9_)	0.21	0.10	1.24	1.01, 1.51	0.036
Downtown (ß_10_)	0.72	0.12	2.05	1.6, 2.64	0.001
**Senior staff**					
Constant (ß_0_)	−0.29	0.07			<0.001
Working time					
4 h (ß_1_)	0.33	0.15	1.39	1.03, 1.86	0.032
3 h (ß_2_)	0.57	0.14	1.76	1.34, 2.34	<0.001
2 h (ß_3_)	1.02	0.16	2.78	2.03, 3.82	<0.001
1 h (ß_4_)	1.49	0.15	4.46	3.32, 5.99	<0.001
Monthly income increment					
15% (ß_5_)	0.34	0.12	1.41	1.11, 1.79	0.005
20% (ß_6_)	0.93	0.14	2.54	1.93, 3.35	<0.001
25% (ß_7_)	0.97	0.13	2.63	2.03, 3.42	<0.001
30% (ß_8_)	1.39	0.29	4.04	2.77, 5.87	<0.001
Working place					
Suburb (ß_9_)	0.47	0.13	1.60	1.25, 2.05	<0.001
Downtown (ß_10_)	0.79	0.16	2.22	1.63, 3	<0.001

Std. Err.: Standard error of coefficient; OR: Odds ratio; 95% C.I.: 95% Confidence interval of OR.

## Data Availability

The data might be achieved by contacting the correspondence author.
